# RNAi spray-induced gene silencing of *EPSPS* by topical application of dsRNA in the weed *Digitaria insularis*

**DOI:** 10.3389/fpls.2025.1688755

**Published:** 2025-10-23

**Authors:** Leonardo A. Cintra, Juliana Da Rosa, Rafael De Assis, Silvana R. R. Marin, Fernando S. Adegas, Elibio L. Rech, Alexandre L. Nepomuceno, Liliane M. Mertz-Henning

**Affiliations:** ^1^ Department of General Biology, Londrina State University, Londrina, Brazil; ^2^ Plant Biotechnology Laboratory, Embrapa Soja, Londrina, Brazil; ^3^ Weed Laboratory, Embrapa Soja, Londrina, Brazil; ^4^ National Institute of Science and Technology in Synthetic Biology, Embrapa Genetic Resources and Biotechnology, Brasilia, Brazil

**Keywords:** SIGS, shikimate-pathway, bioassay, double-stranded RNA, bioherbicide, RNA interference, post-transcriptional silencing

## Abstract

The expanding cultivation of grains to meet agro-industry demands, the implementation of more efficient, sustainable, and integrative management practices capable of mitigating the selection of resistant pests and weeds becomes necessary. Due to the intensive application of chemical herbicides, in addition to environmental impacts, led to the development of resistance mechanisms in weed populations, such as sourgrass (*Digitaria insularis*). These mechanisms complicate management efforts, escalate production costs, and diminish productivity. In this context, RNA interference (RNAi) technology has emerged as a promising molecular tool for the targeted control of weeds, owing to its specificity in the post-transcriptional silencing of vital genes. This study investigated the application of RNAi technology for the suppression of 5-enolpyruvylshikimate-3-phosphate synthase (*EPSPS*) gene expression in *D. insularis*, a weed species of significant agronomic importance in Latin America. A double-stranded RNA (dsRNA) sequence, specifically designed for the *EPSPS* gene, was synthesized via bacterial fermentation with a strain of *E. coli* HT115, extracted using TRIzol™ and purified with phenol: chloroform: isoamyl alcohol, and applied topically in *D. insularis* leaves. The subsequent phenotypic and molecular effects were evaluated. The spray application of the dsRNA resulted in a 44% reduction in the plant's shoot dry mass and a 75% reduction in the number of tillers, thereby indicating consistent physiological impacts due to gene silencing. Quantitative reverse transcription PCR (qRT-PCR) analysis confirmed a significant suppression of *EPSPS* transcript levels following treatment, suggesting partial gene silencing. These findings collectively demonstrate the efficacy of RNAi in modulating gene expression in *D. insularis*, thereby underscoring its potential as a sustainable biotechnological strategy for the development of novel weed control methodologies.

## 1 Introduction

Sourgrass (*Digitaria insularis* (L.) Mez ex Ekman) stands out as one of the most impactful weeds in Brazilian crops. This highly adaptable species fiercely competes for essential resources like water, light, and nutrients (Lopez-Ovejero et al., 2017). It spreads through both seeds and the sprouting of rhizomes, which are underground vegetative structures that store energy, making its control particularly challenging (Ferreira et al., 2019). Due to its high vigor and wide distribution, this species significantly compromises agricultural productivity, especially in grain crops across Latin America (Gazziero et al., 2019). The control of *D. insularis* and other weeds primarily relies on the widespread use of chemical herbicides (Peillex and Pelletier, 2020). However, the intensive and indiscriminate application of these substances has contributed to the selection of tolerant or resistant populations, in addition to causing adverse environmental effects (Lopez-Ovejero et al., 2017; Peillex and Pelletier, 2020).

The extensive adoption of glyphosate in no-tilled fields was as the primary control method for sourgrass for a long time (Correia, 2023). However, over the years, there has been an increase in weed populations that have developed glyphosate resistance, which has become a significant challenge (Galeano et al., 2016). Glyphosate tolerance is primarily linked to target-site factors in the enzyme 5-enolpyruvylshikimate-3-phosphate synthase (*EPSPS*). *EPSPS* is a crucial phosphatase in the shikimate pathway for aromatic amino acid biosynthesis and is the herbicide's target (Almeida et al., 2024). An amplification of the gene copy number can lead to increased enzyme transcription (Patterson et al., 2018), in addition to mutations that alter the active site, leading to resistance (De Carvalho et al., 2012). Furthermore, other non-target-site factors, such as morphological changes and alterations in cellular detoxification metabolism, also contribute to resistance (Comont et al., 2020).

The emergence of populations with multiple resistance and undruggable targets raises an alert for the urgent need to develop new tools for weed control (Heap, 2022; Gao et al., 2025). In this context, new technologies are emerging, focusing on the development of sustainable and effective alternatives for weed management (Dubrovina and Kiselev, 2019). Among these, RNA interference (RNAi) stands out. RNAi is a natural cellular defense mechanism based on the post-transcriptional silencing of genes, present in eukaryotes as a form of cellular defense against viruses and exogenous transposable elements (Fletcher et al., 2020). This pathway is being explored on various fronts, with one of the most promising being the development of formulations for topical application (Spray-Induced Gene Silencing – SIGS), aiming to silencing target genes of interest (Das and Sherif, 2020).

The gene silencing mechanism via RNAi is activated by exogenous double-stranded RNA (dsRNA) molecules. When these enter the plant cell, they're recognized and processed by the DICER enzyme into small interfering RNAs (siRNAs) from 21 to 24 base pairs (Fire et al., 1998; Dalakouras et al., 2016). The siRNAs are then incorporated into the RNA-induced silencing complex (RISC). Its main protein, Argonaute (AGO), uses the siRNA guide strand to recognize and cleave the target messenger RNA (mRNA), leading to its degradation and the subsequent silencing of gene expression (Fletcher et al., 2020).

The consolidation of SIGS technologies in plants faces several bottlenecks, the main one is the efficient delivery to the cells. Plants have multiple leaf barriers, such as the rigid cell wall and selective plasma membrane, which prevent the penetration of large, charged molecules like dsRNAs, in addition to the hydrophobic, waxy cuticle, which is thickened and specialized in grasses (Lopez-Ovejero et al., 2017; Bennett et al., 2020). Considering this background, the present study aimed to prospect the gene encoding the *EPSPS* enzyme, with the goal of designing a specific dsRNA sequence. These dsRNAs were synthesized through bacterial fermentation, purified, and applied topically in a bioassay using sourgrass (*Digitaria insularis*) to evaluate the potential of *EPSPS* gene silencing via RNAi technology.

## 2 Materials and methods

### 2.1 Plant material

Seeds of *D. insularis* of the ecotype named Guaporema (acession number: ID_0903), known for its susceptibility to herbicides, obtained from the seed bank of the Weed Science department at Embrapa Soja (Londrina – PR, Brazil), were sown by spreading in 1-liter pots containing a mixture of soil and substrate in a 3:1 ratio. The germination rate is very high, as expected of grasses, and seedlings were kept in a greenhouse under controlled temperatures ranging from 20°C to 30°C and irrigated twice a day. At 15 days after emergence, thinning was performed to standardize the three tallest seedlings with three fully expanded leaves, not counting the cotyledon, per pot, which were used in the bioassay.

### 2.2 dsRNA application bioassay

To evaluate the effect of dsRNA application on the silencing of the *EPSPS* gene in *D. insularis*, the bioassay was conducted with topical spray application. The vehicle used for application was a 10mM phosphate buffer (pH 6.5), supplemented with 0.1% of the Silwett adjuvant, adapted from Sammons et al. (2011).

Applications were performed early in the morning, using an airbrush calibrated at 15 PSI (1 bar) pressure. Approximately 3mL of the solution, containing the phosphate buffer and the Silwett adjuvant, was applied per treatment. In the specific treatments, dsRNA targeting the *EPSPS* gene was incorporated at a concentration of 100ng·μL^−1^ (**Table 1**). Following application, plants remained unirrigated for 24 hours.

**Table 1:** Experimental design used in the dsRNA and glyphosate application bioassay in *D. insularis*.

Treatment	dsRNA ng.μL^-1^	Carrier	Glyphosate l.ha^-1^	Rep
Control	–	Phosphate Buffer 10mM pH 6,5 + 0,1% Silwett	–	39
dsEPSPS	100		–	39
Glyphosate	–		1,5	39
dsEPSPS + glyphosate	100		1,5	39

Rep, repetition; dsEPSPS, double-strand RNA of EPSPS gene.

Glyphosate herbicide application was carried out 48 hours after dsRNA application, exclusively on the previously defined treatments, at a concentration of 1.5 L·ha^−1^, as suggested by Adegas et al. (2017). After herbicide application, plants again remained unirrigated for 24 hours.

The experimental design adopted was a Completely Randomized Design (CRD), with thirteen replicates per treatment. Each replicate consisted of three plants previously selected for uniformity in height and phenological stage, ensuring the presence of three fully expanded leaves at the time of application.

Five days after the treatments, leaf samples were collected in biological triplicates per treatment and immediately frozen in liquid nitrogen for molecular analyses. Twenty days after applications, tiller counting and shoot collection were performed to determine dry mass. The samples were placed in paper bags, dried in an oven at 56°C for 48 hours, and weighed on a precision analytical balance.

### 2.3 Obtaining and producing dsRNA molecules of the *EPSPS* gene

#### 2.3.1 Extraction and sequencing of the EPSPS gene from sourgrass

Initially, sequences of the *EPSPS* gene from species belonging to the Poaceae family, including *Panicum halli* (GenBank N. XM_025955216.1), *Panicum virgatum* (GenBank N. XM_039986319.1), *Setaria viridis* (GenBank N. XM_034734473.2), and *Setaria italica* (GenBank N. XM_004964389.3), available in the GenBank database (https://www.ncbi.nlm.nih.gov/), were retrieved. The coding sequences (CDS) were aligned using Genefisher2 (Lamprecht et al., 2008) to generate a consensus sequence. After alignment, a phylogenetic tree was constructed with the FastTree software (Price et al., 2010) and edited in iTol (Letunic and Bork, 2021).

For genomic DNA extraction, leaf samples of *D. insularis* collected in liquid nitrogen were stored in a –80°C freezer to preserve the nucleic acids for subsequent extraction. The genomic DNA was extracted using the CTAB buffer (Cetyltrimethylammonium Bromide), following the protocol of Doyle and Doyle (1987). DNA quantification was performed by spectrophotometry using Nanodrop (Thermo Scientific), while integrity was verified by 1% agarose gel electrophoresis.

The consensus sequence was used as a template for designing specific primers using the Primer3Plus software (https://www.bioinformatics.nl/cgi-bin/primer3plus/primer3plus.cgi), as listed in **Table 2**. The primers were used for amplifying fragments of the *EPSPS* gene from *D. insularis* using genomic DNA as template. The generated amplicon was validated through sequencing, aiming to find correspondence with the set of CDS used. For this, the High-Fidelity PCR reaction was performed with template DNA in a final volume of 50 μL, containing 2.0 mM MgSO_4_, 200 μM of each dNTP, 0.2 μM of each primer, and 1.5 U of High-Fidelity Taq DNA polymerase.

**Table 2:** Primers for sequencing, RT-qPCR, and qPCR analysis of the *EPSPS* gene from Sourgrass.

Objective	Gene	Sequence 5’-3’	TA (°C)	Amp. (bp)
Sequencing	*EPSPS*	F- GCTGGTGGAAATGCAACTTA	52	440
		R- GAAATAGCTTGCACTTGAGG	52	
RT-qPCR	*EPSPS*	F – CAACCGCAATCAGAGATGTG R - GGACCTTCCTCAACTGATGC	60 60	106
	*2APP*	F- TGGTCTGATCCAGATGATCG R- CCCGGGCTACAAGTTTAAGA	60 60	121
	β-Tub	F- AGATGTGGGATGCCAAGAAC R- TCTTGTTCTGCACGTTGAGC	60 60	80
Cloning	*EPSPS*	F-GGGCGTCTCGCGAATCTATCTGG TTCGATCAGCAG	58	191
		R-GGGCGTCTCGAGACCTTGATGCA GAACCTGTCC	58	

F, foward; R, reverse; TA, temperature of annealing; Amp, amplicon; bp, bases pair.

The amplification conditions consisted of an initial denaturation step at 95°C for 5 minutes, followed by 35 cycles of 30 seconds at 94°C, 30 seconds at the specific annealing temperature for each primer pair (**Table 2**), and 40 seconds at 68°C, finalizing with a final extension of 7 minutes at 72°C. The amplified products were verified by 1% agarose gel electrophoresis and subsequently purified for sequencing. Sequencing was conducted using the BigDye™ Terminator v3.1 Cycle Sequencing kit (Applied Biosystems), according to the manufacturer's specifications, on an ABI3500 (Applied Biosystems) equipment.

#### 2.3.2 Obtaining and producing dsRNA molecules of the *EPSPS* gene via fermentation

The fragments obtained by sequencing the *EPSPS* gene were analyzed using the siRNA Wizard software (https://www.invivogen.com/sirnawizard). The goal was to identify regions with the highest potential for generating siRNAs, based on the preferential cleavage sites of the DICER enzyme. From this analysis, a region with the highest probability of effectiveness for gene silencing was selected (**Table 3**), and submitted to an off-target analysis contrasting the fragment using BLASTn with the genomes of three key-species (*Zea mays*, *Triticum aestivum* and *Glycine max*). The dsRNA sequence was aligned with the gene for annotation using the IBS 2.0 software (Lamprecht et al., 2008; Xie et al., 2022).

**Table 3:** dsRNA sequence used in topical applications in the bioassay.

Id	Sequence 5’ – 3’	Size (bp)
EPSPS	CUAUCUGGUUCGAUCAGCAGUCAAUACUUGAGCGCCUUGCUGAUGGCUGCUCCUUUAGCUCUUGGGGAUGUGGAGAUUGAGAUCAUUGAUAAACUAAUCUCCAUUCCCUAUGUCGAAAUGACAUUGAGAUUGAUGGAGCGUUUUGGCGUGAAAGCAGAGCACUCUGAUAGCUGGGACAGGUUCUGCAUCAA	191

Bp, bases pair.

The synthesis of the dsRNA targeting the *EPSPS* gene (dsRNA-EPSPS) was carried out at the Plant Biotechnology Laboratory of Embrapa Soybean. This was achieved through bacterial expression in *Escherichia coli* HT115 (DE3), transformed with pClone_VR vector containing the insert of dsRNA – EPSPS (**Table 3**), as described by Da Rosa et al. (2024). The pClone_VR vector was assembled with the insert after digestion with restriction enzymes BsmBI (Anza™), performing a ligation in 5’- 3’ sense of the insert using T4 DNA ligase in the multiple cloning site (MCS) flanked by bidirectional T7 promoters and terminators. Bacterial transformation was performed by heat shock, followed by cultivation of the recombinant colonies in liquid LB medium (500 mL), supplemented with tetracycline (12.5 μg·mL^−1^) and ampicillin (100 μg·mL^−1^) antibiotics.

Induction of dsRNA expression was conducted by adding lactose, maintaining the culture under agitation at 180 rpm at 37°C for a period of five hours. After this time, cells were centrifuged at 4000 g for 5 minutes, and the bacterial pellet was subjected to total RNA extraction using TRIzol™ reagent, as described by Da Rosa et al. (2024). Subsequently, the RNA was purified by treatment with 2 μL DNase (Ambion) and 20 μL RNase A (Invitrogen) to remove DNA and single-stranded RNA.

The purification was performed adding 200 μL of phenol: chloroform: isoamyl alcohol (25:24:1), and after vigorous stirring, centrifugation was performed at 12 000 ×g for 15 min. The upper phase was transferred to another tube, 100 μL of NH_4_OAc (7.5 M) and 100 μL of isopropanol were added, and the samples were centrifuged at 12 000 ×g for 45 min at 4°C. This step was followed by two washes with 70% ethanol and centrifugation at 12 000 ×g for 10 min. The pellet was dried and resuspended in nuclease-free water, as described by (Ahn et al., 2019).

The purified dsRNA was quantified by spectrophotometry using a Nanodrop (Thermo Scientific) at 260 nm, and evaluated for integrity and expected size by 1% agarose gel electrophoresis (**Supplementary Figure 1**).

### 2.4 Gene expression analysis by RT-qPCR

#### 2.4.1 RNA extraction and cDNA synthesis

The extraction of total RNA was conducted following the protocol established by Oliveira et al. (2015). RNA quantification and purity were evaluated using a Nanodrop spectrophotometer (Thermo Scientific), and its integrity was analyzed by 1% agarose gel electrophoresis. To eliminate possible genomic DNA contaminants, RNA samples were treated with DNase I (Amplification Grade, Invitrogen, Thermo Scientific), following the manufacturer's instructions.

cDNA synthesis was performed from messenger RNA (mRNA) using the SuperScript III First-Strand Synthesis System for RT-PCR (Invitrogen, Carlsbad, CA, USA) kit, according to the manufacturer's protocol. The efficiency of the DNase I treatment and cDNA synthesis were evaluated by PCR with the same specific primers for the EPSPS gene (**Table 2**), followed by verification on a 1% agarose gel.

#### 2.4.2 Gene expression analysis

RT-qPCR reactions were performed in technical triplicates on a 7900HT Fast Real-Time PCR System (Applied Biosystems) using the Platinum® SYBR Green® qPCR SuperMix-UDG with ROX (Invitrogen) kit, according to the manufacturer's specifications. The primers were designed with Primer3Plus software (**Table 2**). The amplification protocol consisted of an initial denaturation step at 95°C for 10 minutes, followed by 40 cycles of 95°C for 15 seconds and 60°C for 1 minute for annealing and extension. At the end, a dissociation curve analysis was performed with a gradual temperature increase up to 95°C for 15 minutes, followed by cooling to 60°C for 1 minute.

The primers efficiency for quantifying *EPSPS* gene expression was determined from standard curves generated with serial dilutions of cDNA (1:5, 1:25, 1:125, 1:625, 1:3125). The efficiency index (E) was calculated using the formula: E = 10^−1/slope^, based on the slope of the curve. The amplified products specificity was verified by analyzing the peaks of the dissociation curves.

Relative *EPSPS* gene expression was quantified using the 2^−ΔΔCt^ method described by Livak and Schmittgen (2001). For data analysis and normalization, the reference genes Serine/Threonine-phosphatase (2APP) and β-Tubulin (β-Tub) were used as calibrators, whose primers are listed in **Table 2**, and were previously identified in *D. insularis* (Da Rosa, unpublished data).

#### 2.4.3 Statistical analyses

The data collected in the experiments were subjected to analysis of variance (ANOVA), after verifying the assumptions of normality by Shapiro-Wilk test, homogenity of variances by Bartlett test, and independence of the residuals by Durbin-Watson test. The means were analyzed by Duncan's test, with α < 0.05. For comparison between two factors, the assumption of normality was first verified using the Shapiro-Wilk test. As the data did not follow a normal distribution, the Mann-Whitney U test was applied to assess the difference between the groups (p < 0.05).

## 3 Results

### 3.1 Prospecting and biosynthesis of dsRNA-*EPSPS*

**Figure 1:**
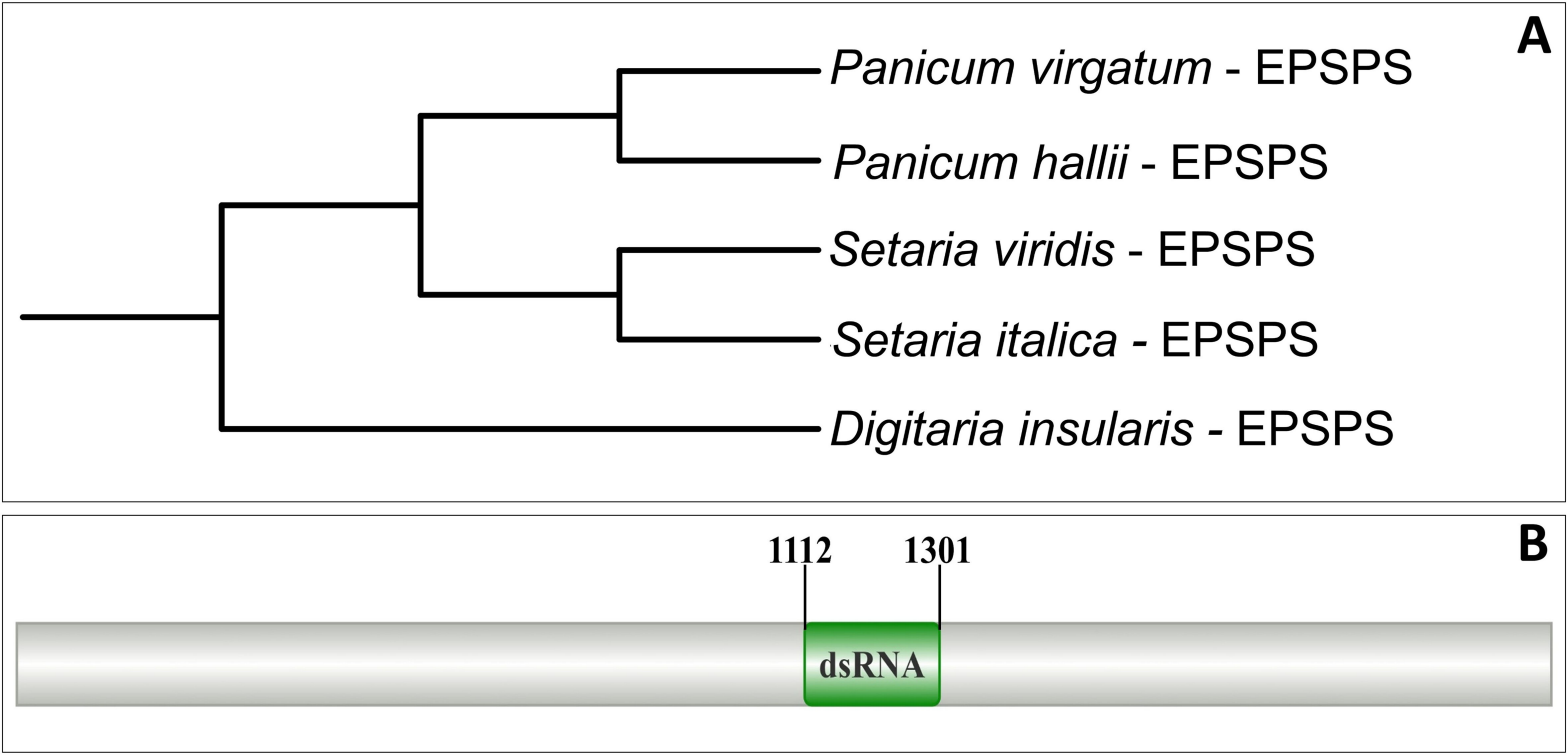
Strategy for prospecting the dsRNA for the *EPSPS* gene. **(A)** Phylogenetic tree constructed from the *EPSPS* gene sequences of Poaceae species. **(B)** Annotation of the position of the prospected dsRNA under the *EPSPS* gene for silencing via RNAi.

Multiple sequence alignment analysis of the *EPSPS* gene from phylogenetically related species revealed a well-conserved fragment of approximately 1100 bp (**Supplementary Figure 2**), which made possible the construction of a phylogenetic tree (**Figure 1A**) and the design of primers (**Table 2**). The sequencing reads were used for the reconstruction of a fragment of approximately 420 bp of the *EPSPS* gene, which was employed in the selection of the dsRNA target region (**Figure 1B**) and the design of primers for cloning and qRT-PCR (**Table 2**).

This made it possible to proceed with the assembly of the pClone_VR vector with the and the production of dsRNA via bacterial fermentation, using *E. coli* HT115 strain properly transformed. After purification the dsRNA reached 2707 ng·μL^−1^ in spectrophotometry, and approximately 100 ng·μL^−1^ of dsRNA was obtained and applied in each treatment of the bioassay. Molecular results of the production and a schematic design of the pClone_VR vector are presented in **Supplementary Figure 3**.

### 3.2 Topical dsRNA application bioassay

**Figure 2:**
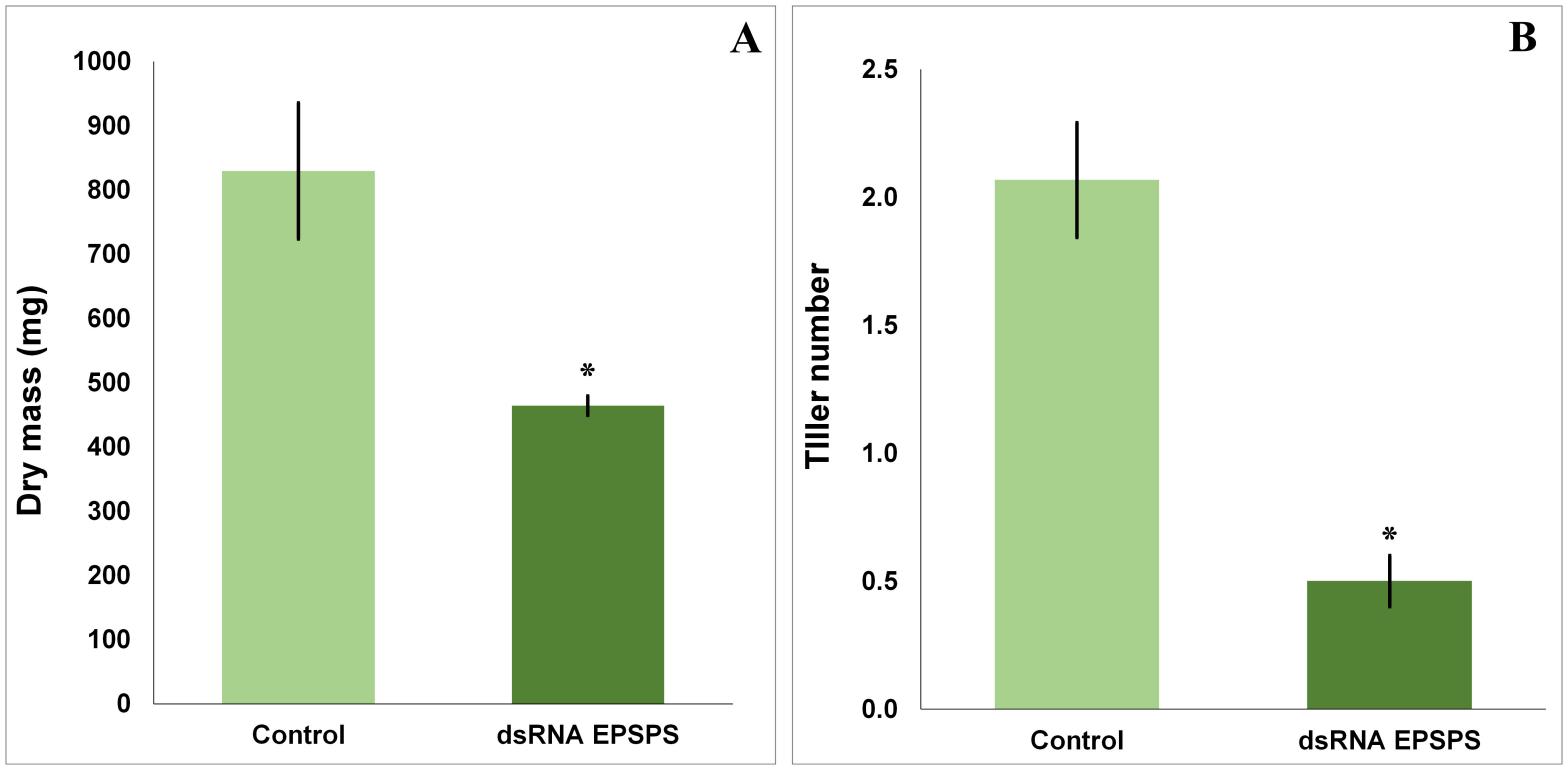
Dry mass and tiller number results obtained after evaluation and collection of *D*. *insularis* treated in a bioassay. **(A)** Mean dry mass of the treatments (n=10 per treatment). **(B)** Mean number of tillers of the treatments (n=30 per treatment). The data in the graphs are means ± standard deviation. “*” indicates a significant difference by Mann-Whitney T test, *p* < 0.05, with a significant decrease in both.

Topical application of dsRNA specific to the *EPSPS* gene in *D. insularis* promoted significant phenotypic changes in the treated plants. The group exposed exclusively to dsRNA, an average reduction of 44% in shoot dry mass (**Figure 2A**) was observed, accompanied by a significant decrease of 75% in the number of tillers (**Figure 2B**) when compared to the control group and as analyzed by the Mann-Whitney T test (p < 0.05). These effects indicate a direct physiological impact related to the partial silencing of the target gene.

Treatments where glyphosate was applied at the commercial dose of 1.5 L·ha^−1^ resulted in a lethal effect on the plants as early as 7 days after application. This effect was expected, given the physiology of the herbicide-susceptible ecotype used in the analyses of this study. The herbicide response culminated in the absence of biomass and tillering in the replicates of this experimental group, making the collection of subsequent quantitative data impossible. Therefore, no results from these treatments are shown in **Figure 2**.

### 3.3 Relative expression of the *EPSPS* gene after bioassay

**Figure 3:**
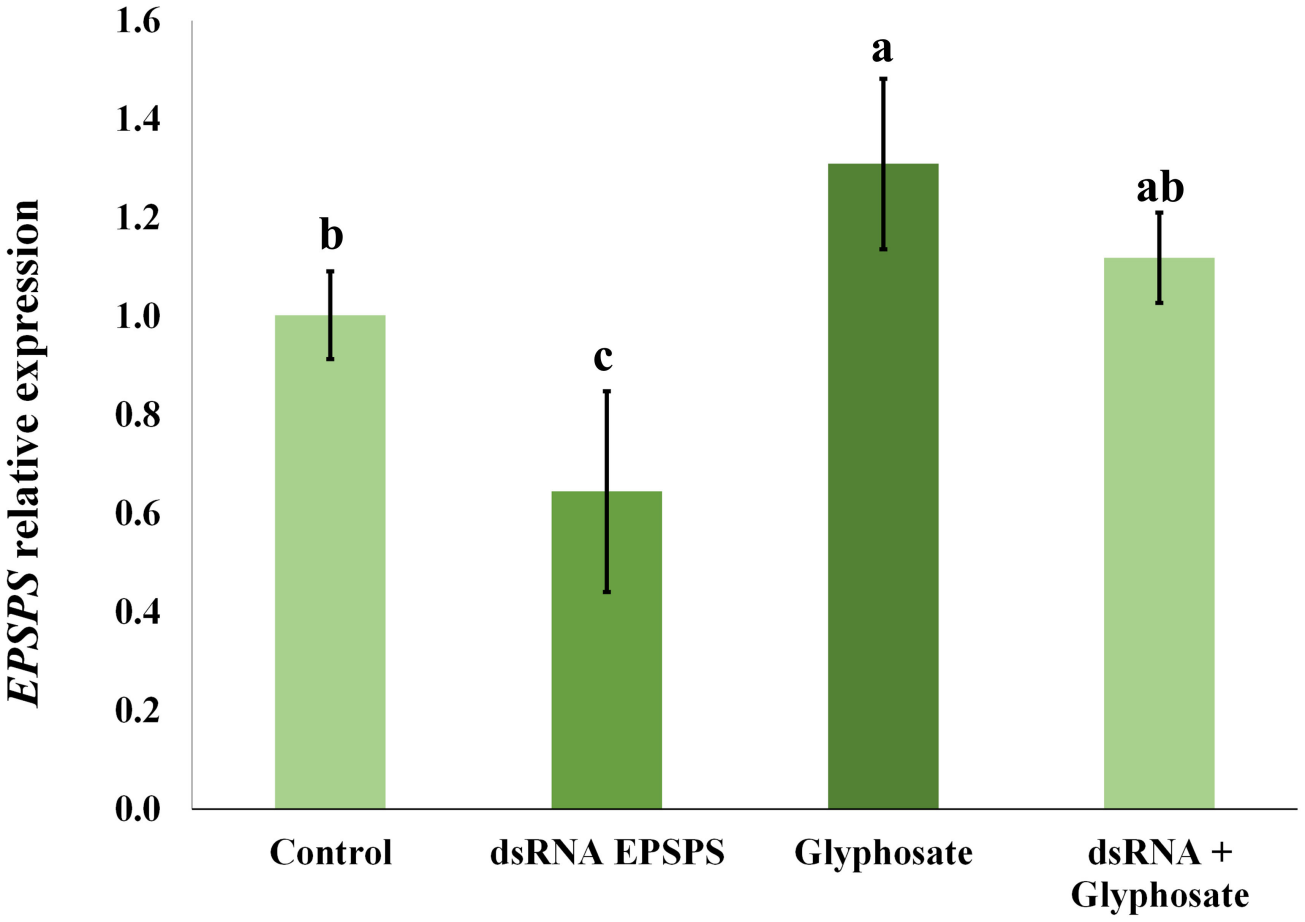
Relative expression of the *EPSPS* gene of *D. insularis* after bioassay treatment. The letters above the bars indicate the differential variance observed in each treatment. A low regulation of transcripts is observed in the dsRNA EPSPS treatment, while there is an increase in transcripts in the glyphosate treatment and stability in the dsRNA + glyphosate combination. The data in the bar chart are means ± standard deviation. The data were subjected to subjected to an ANOVA variance test (F = 10.63; p = 0.0036) followed by Duncan's test, *p* < 0.05. The coefficient of variation (CV) was 14,59%. b: 1.001433, c: 0.644480, a: 1.309267, ac: 1.115660.

With samples collected 5 days after the bioassay application, it was possible to perform the RT-qPCR analysis. A significant reduction in the relative expression of the *EPSPS* gene was observed in *D. insularis* treated with dsRNA, reaching 36% of the expression level compared to the control treated only with the vehicle solution (**Figure 3**). The difference was statistically significant, as analyzed by variance test ANOVA (**Supplementary Figure 4**) and *post-hoc* Duncan's test (p < 0.05), indicating that the silencing by RNAi technology was partial.

In addition, a significant increase in *EPSPS* transcripts was detected in the treatment where glyphosate was applied alone (**Figure 3**), demonstrating an adaptive transcriptional response to the stress caused by the herbicide. However, the combined application of dsRNA and glyphosate showed no change in relative expression, indicating that the adaptive transcriptional response compensated for the silencing promoted by RNAi. The collection of glyphosate-application data was only possible because the plants are still alive in the time of five days after herbicide application.

The partial reduction of *EPSPS* gene expression by RNAi observed in this study, in addition to the phenotypic effect of reduced dry mass and tillering, demonstrates the potential of the technology for the development of solutions based on topical biodirected herbicides, or SIGS (Spray-Induced Gene Silencing).

## 4 Discussion

### 4.1 Prospecting and biosynthesis of dsRNA-EPSPS

Despite the agronomic relevance of *D. insularis*, genomic and transcriptomic data for this species are still scarce in public databases (Montgomery et al., 2024). Choosing conserved regions of the *EPSPS* gene from multiple alignments among species of the same family represents an effective strategy for gene prospecting, especially for those involved in essential metabolic pathways whose functional integrity is critical, such as *EPSPS* in the shikimate pathway. Studies such as those by Rafique et al. (2023) show that the use of orthologous sequences permits the identification of ideal segments for primer design and the selection of dsRNA target regions with good specificity.

The production of dsRNA via a bacterial fermentation process is a consolidated and relatively low-cost alternative for the large-scale generation of molecules for agricultural application (Mezzetti et al., 2020). This technology has proven promising, especially when combined with purification methods that ensure the absence of contaminants, in addition to the use of lactose as an inducer to optimize production and the cost of biosynthesis (Da Rosa et al., 2024). Additionally, the feasibility of this approach for topical applications has been demonstrated in real-world systems, such as in the work of Rafique et al. (2023) with *Triticum aestivum*, pointing to a viable path for the development of RNAi-based bioherbicides.

### 4.2 Topical dsRNA application bioassay

The phenotypic results of dry mass and tillering observed in the bioassay indicate the action of dsRNA in gene silencing, directly interfering with the plant's vegetative development. This likely occurs due to the limitation caused in the synthesis of aromatic amino acids mediated by the shikimate pathway (Almeida et al., 2024), similar to what is observed with inhibitors that act on this pathway.

The bioassay results corroborate the data presented by Lau et al. (2015) with *Dendrobium hybrid*, Kiselev et al. (2021) with *Arabidopsis thaliana*, and even in other weed species, such as Sammons et al. (2011) and (2018) with *Amaranthus palmeri*, Hendrix et al. (2021) with *Amaranthus cruentus*, and Mai et al. (2021) with *Mikania micrantha*. These authors successfully silenced the endogenous genes *R2R3-MYB, CHS, MYBL2, ANAC032, PDS, EPSPS, CHL-I, CHL-H, GUN-4*, and *CAP10A* via topical RNAi application, and even observed phenotypic effects of the silencing after bioassay treatment, similar to what was observed in *D. insularis* in this study.

The difference lies in the formulation of the vehicle solution for application. Sammons et al. (2011) and (2018) used Silwett-L77 as an adjuvant, as was done in this study. The surfactant effect of this class of substances is what allows for better spreading and absorption of the dsRNA molecules in the leaf tissues, aiding in the transposition of barriers like the cuticle and cell wall (Bennett et al., 2020), which are responsible for the plant's defense against exogenous agents.

### 4.3 Relative expression of the *EPSPS* gene after bioassay

Topical application of dsRNA resulting in partial silencing of the *EPSPS* gene in *Digitaria insularis* is in line with evidence from the literature that RNA interference (RNAi) technology can be effective in regulating essential genes in agricultural plants (Panozzo et al., 2025). Previous studies, such as those by Sammons et al. (2011) and (2018), Hendrix et al. (2021), and Mai et al. (2021), have demonstrated success in reducing gene expression by RNAi via exogenous dsRNA application, and also partial downregulation like in this study. This partial effect on silencing is discussed by the literature as being caused by deficiencies in the delivery and proper processing of dsRNA by dicer (Panozzo et al., 2025), but molecular improvements may increase silencing efficiency to validate this approach as a viable strategy in weed species.

In the present study, in addition to the reduction of relative *EPSPS* expression, an increase in gene expression was observed when the plants were exposed to glyphosate, which suggests a mechanism of transcriptional upregulation induced by chemical stress, as previously reported to *EPSPS* gene and other genes from shikimate pathway in the weed *A. palmeri* susceptible ecotype treated with glyphosate (Zulet-González et al., 2020). This type of adaptive response is explained by the transient lack of Aromatic Amino Acids (AAA) and the accumulation of shikimate as a regulatory signal (Zulet-González et al., 2020). It has already been documented in other weed species, such as in *Conyza bonariensis* (Hereward et al., 2018), reinforcing the relevance of *EPSPS* as a strategic target for overcoming the challenges of glyphosate resistance.

Despite the evident potential of RNAi for the targeted control of weeds, one of the main challenges for its large-scale application lies in optimizing the delivery process of dsRNA molecules. The effectiveness of gene interference is directly related to the stability of the dsRNA, its ability to penetrate plant tissues, and its functional integrity until it reaches the target mRNA (Yang et al., 2022). The mechanism of cellular uptake of dsRNA molecules in plants is still in discussion by the recent literature, De Schutter et al. (2022a) describes the cellular uptake *in vitro* of a 500 bp dsRNA, but only in *A. thaliana* cells. The lack of information is caused by the difficulty in reproducing and implementing cellular uptake assays in plants, because its specialized cell wall and a very selective membrane, which both represent important physical barriers for dsRNA uptake, especially in the case for long molecules like dsRNA (Bennett et al., 2020; De Schutter et al., 2022a).

The partial silencing obtained in our study could be overcome by using strategies that increase the silencing potential and delivery efficiency of the dsRNA molecule (Yan et al., 2021). Recent advances have pointed out promising alternatives, especially through nanotechnology applied to delivery. Among the approaches, the use of nanoparticles for protection and peptides as carriers (Pepper et al., 2017; Schwartz et al., 2020) stands out, in addition to direct chemical modifications to dsRNA molecules, which can increase their resistance to degradation by nucleases and facilitate their internalization in plant cells (Hunter and Wintermantel, 2021; Yan et al., 2021).

Additionally, the consolidation of RNAi as a commercial agricultural technology faces technical and scientific bottlenecks, such as the scarcity of genomic data in weed species, difficulties in formulating stable products, and, above all, the feasibility of efficient delivery mechanisms to the site of action (Baulcombe, 2004; Bennett et al., 2020; De Schutter et al., 2022b).

To date, only one RNAi-based product has been officially launched on the market: "Calantha," developed by GreenLight Biosciences for the control of the Colorado potato beetle (*Leptinotarsa decemlineata*) (Rodrigues et al., 2021). There are many different bottlenecks in RNAi approaches between plants and metazoans, and the main one is the greater ease of validating target genes and delivery strategies, as metazoans do not have as many barriers that prevent direct topical application as plants do (Panozzo et al., 2025). The formulation, based on the topical application of the dsRNA "Ledprona," represents the first case of industrial validation of the effectiveness of gene silencing as an alternative to the use of conventional insecticides (Rodrigues et al., 2021), and serves as a model for future initiatives and validation of RNAi technology.

## 5 Conclusion

The results from this study demonstrate that the topical application of double-stranded RNA (dsRNA) targeting the *EPSPS* gene partially silenced gene expression in *D. insularis*. This led to measurable phenotypic impacts, including reductions in dry mass and tiller count. The expression of the *EPSPS* gene was significantly reduced after dsRNA application, confirming the action of the RNAi mechanism in the studied species. These findings strengthen the viability of RNAi technology as a molecular strategy for the targeted control of agriculturally relevant weeds.

The observed transcriptional response also revealed the induction of *EPSPS* expression in the presence of herbicide. This corroborates the gene's regulatory role in response to chemical stress and underscores the relevance of its silencing as an integrative management tool. Thus, this work provides important experimental evidence for the consolidation of RNAi as a promising biotechnological approach, paving the way for the development of new-generation herbicides based on the targeted silencing of genes in weeds.

## Data Availability

The original contributions presented in the study are included in the article/**Supplementary Material**. Further inquiries can be directed to the corresponding author/s.
