# Supplementary material for: RNAi spray-induced gene silencing of EPSPS by topical application of dsRNA in the weed Digitaria insularis
Source: Front Plant Sci. 2025 Oct 23;16:1688755. doi: 10.3389/fpls.2025.1688755 (PMC12589023; doi:10.3389/fpls.2025.1688755)
Supplement: Supplementary file 1 [file SupplementaryFile1.docx]

# Supplementary


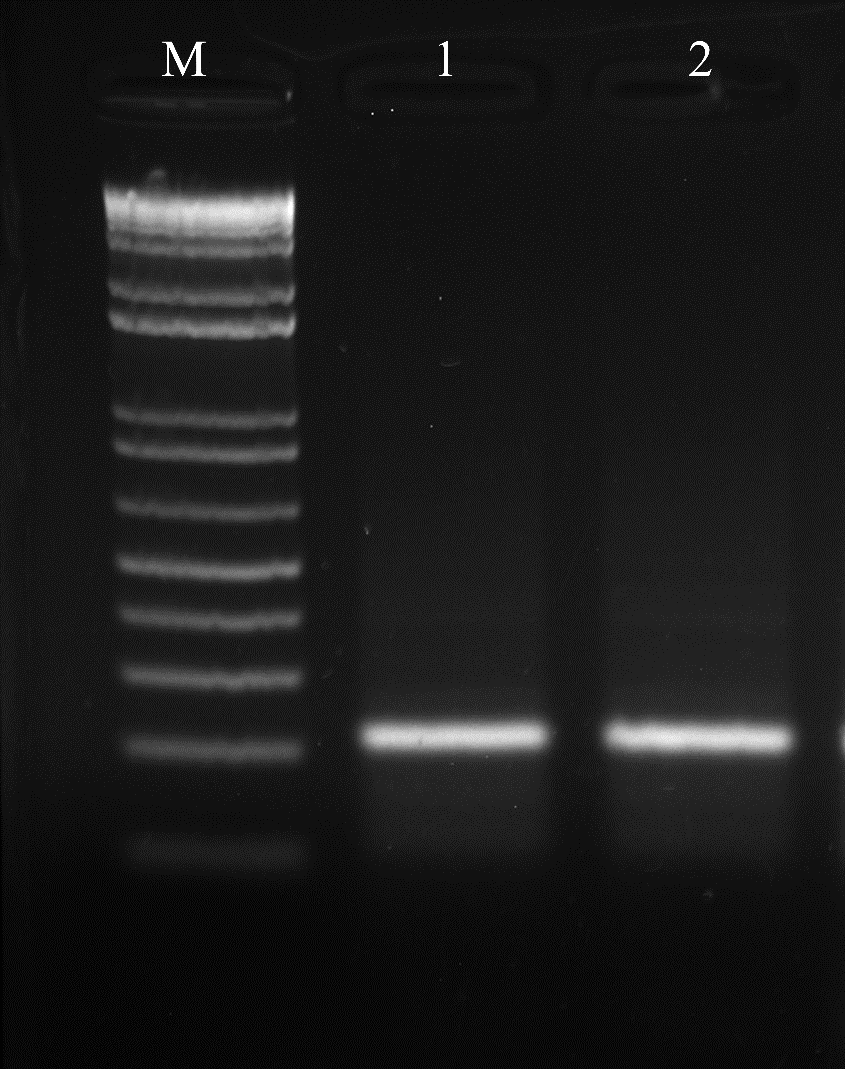


**Supplementary 1.** Production of dsRNA from EPSPS gene using the pCloneVR_2 and produced with *E. coli* HT115 fermentation. M: 1 kb plus DNA ladder; 1 and 2: purified dsRNA from EPSPS gene.


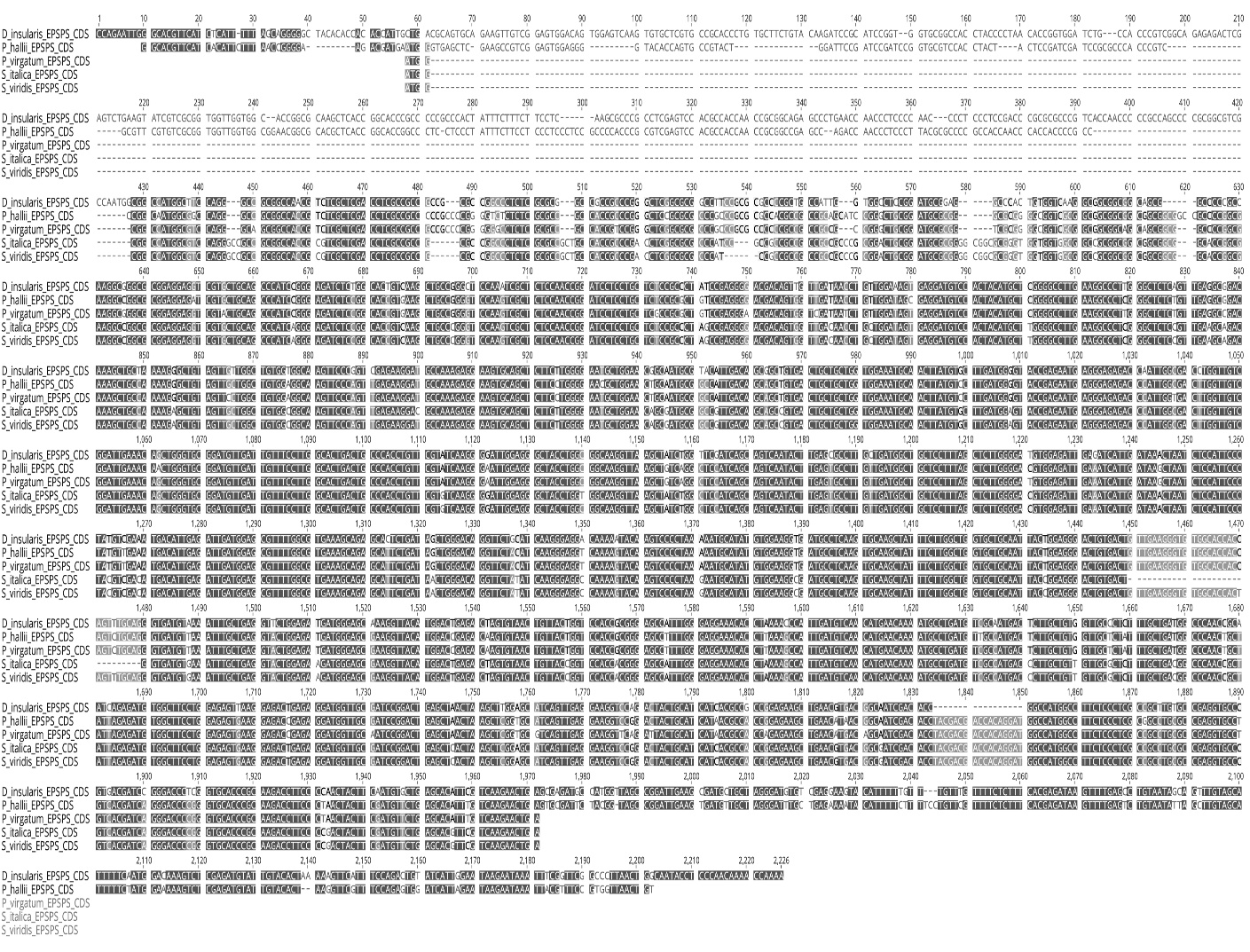


**Supplementary 2.** Multiple alignment of the *EPSPS* gene sequence from *Digitaria insularis*, *Panicum hallii*, *Panicum virgatum*, *Setaria italica* and *Setaria viridis* generating a consensus sequence of *EPSPS* gene. The shaded bases represent greater similarity (100%) between genes, and the lines “-“ represent gaps.


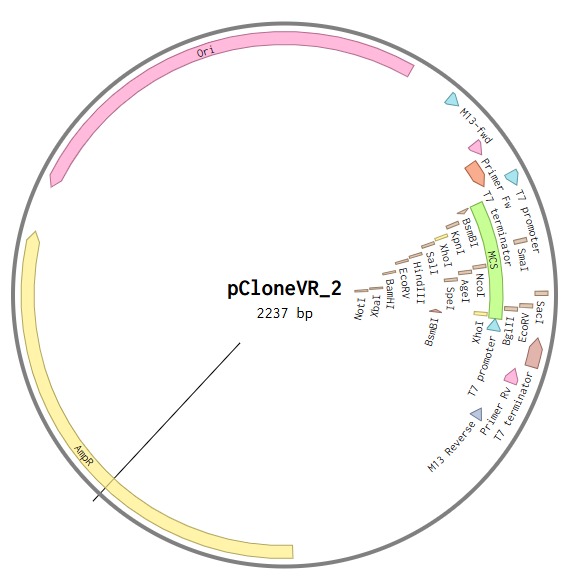


**Supplementary 3.** Scheme of pCloneVR_2 showing the multiple cloning site flanked by bidirectional T7 promoters and terminators. Construction of the expression vector: AmpR: Ampicillin Resistance (ampicillin resistance gene), BamHI: *Bacillus amyloliquefaciens* H (restriction enzyme), BglII: *Bacillus globigii* II (restriction enzyme), ClaI: *Corynebacterium lilium* A (restriction enzyme), EcoRV: *Escherichia coli* RV (restriction enzyme), HindIII: *Haemophilus influenzae* Rd III (restriction enzyme), KpnI: *Klebsiella pneumoniae* I (restriction enzyme), M13: Single-stranded filamentous bacteriophage M13 (primer sequence derived from the phage), MCS: Multiple Cloning Site, NcoI: *Nocardia corallina* I (restriction enzyme), NotI: *Nocardia otitidiscaviarum* I (restriction enzyme), Ori: Origin of replication, PstI: *Providencia stuartii* I (restriction enzyme), SacI: *Streptomyces achromogenes* I (restriction enzyme), SalI: *Streptomyces albus* I (restriction enzyme), SpeI: *Serratia marcescens* I (restriction enzyme), T7: Bacteriophage T7 (promoter or terminator derived from this phage), XbaI: *Xanthomonas badrii* I (restriction enzyme), XhoI: *Xanthomonas holcicola* I (restriction enzyme).


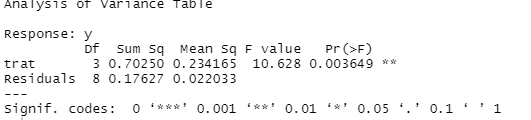


**Supplementary 4.** Analysis of variance (ANOVA) table. F = 10.628; Freedon degree of treatments = 3 freedon; degree of residuals = 8; p-value = 0.003649. Independence Durbin-Watson test (p-value 0.516). Shapiro-Wilk normality test (p-value = 0.3013). Bartlett test for variance homogeneity (p-value 0.8235)
